# Depth Estimation for Light-Field Images Using Stereo Matching and Convolutional Neural Networks

**DOI:** 10.3390/s20216188

**Published:** 2020-10-30

**Authors:** Ségolène Rogge, Ionut Schiopu, Adrian Munteanu

**Affiliations:** Department of Electronics and Informatics, Vrije Universiteit Brussel, Pleinlaan 2, 1050 Brussels, Belgium; ischiopu@etrovub.be (I.S.); acmuntea@etrovub.be (A.M.)

**Keywords:** depth estimation, stereo matching, convolutional neural networks, light-field images

## Abstract

The paper presents a novel depth-estimation method for light-field (LF) images based on innovative multi-stereo matching and machine-learning techniques. In the first stage, a novel block-based stereo matching algorithm is employed to compute the initial estimation. The proposed algorithm is specifically designed to operate on any pair of sub-aperture images (SAIs) in the LF image and to compute the pair’s corresponding disparity map. For the central SAI, a disparity fusion technique is proposed to compute the initial disparity map based on all available pairwise disparities. In the second stage, a novel pixel-wise deep-learning (DL)-based method for residual error prediction is employed to further refine the disparity estimation. A novel neural network architecture is proposed based on a new structure of layers. The proposed DL-based method is employed to predict the residual error of the initial estimation and to refine the final disparity map. The experimental results demonstrate the superiority of the proposed framework and reveal that the proposed method achieves an average improvement of 15.65% in root mean squared error (RMSE), 43.62% in mean absolute error (MAE), and 5.03% in structural similarity index (SSIM) over machine-learning-based state-of-the-art methods.

## 1. Introduction

Light-field (LF) cameras were recently introduced in the image-processing and computer-vision domains in order to resolve the limitations of the conventional camera model. Conventional cameras, which capture the red, green and blue (RGB) primary colours, were designed to capture the color and the accumulated light intensity of the incoming light rays from all directions incident to the camera plane at each pixel position. In contrast to this model, LF cameras were designed to capture the intensity, color, and directional information of each light ray at each pixel position, yielding a 4D LF image for each acquisition.

LF cameras, also known as plenoptic cameras, are implemented by placing an array of microlenses in front of the camera sensor. They serve as an alternative to the conventional paradigm to acquire 4D LF data, which is to arrange conventional RGB cameras on a rectangular grid. Conventional camera systems are difficult to implement and handle, and the inherently large baselines between cameras yield substantial difficulties when handling occlusions in many applications.

The advantages brought by LF cameras were recently demonstrated in several image-processing applications such as depth estimation [1,2,3,4,5], refocusing [6], calibration [7,8], editing [9], matting [10], face analysis [11], and 3D point cloud enhancement [12], to name a few.

The problem of depth estimation from stereo images was widely investigated during several decades. Recently, LF images have received more attention due to their capability to provide both light ray intensity and directional information about the scene. Stereo matching was limited to the correspondence cue, whereas a LF image can also provide defocus and shading cues [13,14]. However, several issues must be addressed to achieve optimal depth estimation from LF images. The depth-estimation problem for LFs is equivalent to estimating depth in multi-camera systems. Since a LF image can be represented as a camera array of sub-aperture images (SAIs) with narrow baselines, finding matching correspondences between the SAIs remains a very challenging task as it is difficult to achieve robust disparity estimates with high precision for such small displacements. However, one can refine the final depth information by fusing the passive depth maps estimated from light-field data with the active depth maps captured by depth sensors. Works such as [15,16,17,18] would benefit from an accurate depth model as the accuracy of their reconstructed 3D models is of utmost importance. Since the quality of depth estimation influences the overall quality of subsequent processing steps, the goal of this paper is to provide an efficient depth-estimation model to improve the 3D reconstruction in applications such as underwater 3D reconstruction [19], 3D printing [20], or building renovation [21].

Even though LFs provide more information about the scene compared to a regular RGB image, depth estimation based on LF images still remains a challenging task. Most recent state-of-the-art algorithms, such as [22,23], propose to employ Machine-Learning (ML) techniques to improve over traditional computer-vision-based algorithms. The multi-stereo matching techniques tend to provide a more robust performance; however, their depth-estimation results are affected by different types of distributions of the depth-estimation errors: in the flat areas due to depth uncertainties, and around the edges due to disocclusions. The goal of this paper is to propose a depth-estimation algorithm for LF images which combine these two techniques by allying the robustness of the computer-vision algorithms and the efficiency of deep learning-based (DL) algorithms. In this paper, a multi-stereo matching algorithm is first employed on a corresponding set of different pairs of SAIs in the LF image to generate the initial disparity map of the central view. The initial estimation is then refined by employing a DL-based algorithm for residual error prediction.

In our prior work [24,25] we tackled the problem of depth estimation using both multi-stereo matching techniques and ML techniques. In [24], we proposed a depth-estimation method based on the multi-scale multi-window approach which employs belief propagation to regularize the results. In [25], we proposed a DL-based depth-estimation method, where a neural network is employed to compute the disparity of each pixel by processing the 3D block patches extracted from epipolar plane images (EPIs). In this paper, we first extend our algorithm from [24] and employ it to compute an initial depth estimation, and then we follow a residual error prediction approach where a novel DL-based algorithm is employed to refine the initial estimation. The proposed approach was inspired by our previous work for DL-based lossless image compression [26,27], where a DL-based dual prediction method is employed to compute an improved prediction.

The contributions of this paper are as follows: (a) a novel comprehensive framework for depth estimation which combines stereo matching and ML techniques; (b) an improved stereo matching algorithm designed to operate on any SAI pair; (c) a novel neural network architecture for depth residual error prediction based on a new and efficient block structure; and (d) an efficient depth-estimation method with outstanding performances when compared with state-of-the-art methods.

The rest of the paper is organized as follows. Section 2 provides an overview of the existing depth-estimation techniques for LF images. Section 3 describes the proposed method based on stereo matching and ML techniques. Section 4 presents the experimental validation. Section 5 draws the final  conclusions.

## 2. Related Work

The existing techniques in the field of depth estimation for LF cameras can be divided into two categories: (i) conventional computer-vision methods; and (ii) DL-based methods.

### 2.1. Conventional Computer-Vision Methods

The rise of LF cameras allows the use of different cues, as it is not needed to shoot multiple images separately to capture the scene with various focus or view point anymore. Thus, Tao et al. [28] devised an algorithm to compute a dense depth estimation by combining multiple cues, namely defocus and correspondence. This work was extended in [29] by refining the shape using the shading cue, and by removing the specularity component of a scene, allowing for better depth estimation in specular areas. Wang et al. [3] developed a depth-estimation algorithm focusing on the main issues of [28] by detecting and improving the depth of occluded regions. Back to the correspondence cue, Jeon et al. [4] estimated the depth by shifting SAIs using the phase shift theorem, the gradient costs and a multi-label optimization, while Buades et al. [30] combine multiple pairwise disparity estimations using a multi-scale and multi-window stereo matching algorithm which rejects the unreliable pixels. Navarro and Buades [31] proposed to improve the disparity estimations of [30] by employing an optical-flow algorithm to interpolate the disparity. Williem et al. [32] proposed two new data costs to improve the depth estimation in noisy and occluded areas, where the first one is based on the angular patch approach and the second on the refocus image approach. Huang [33] developed a stereo matching algorithm by employing an empirical Bayesian framework based on Markov Random Fields to infer the depth maps of any kind of scenes—dense, sparse, denoised, RGB or grayscale.

A different approach is based on the estimation of the slopes of an epipolar plane image (EPI) to compute the depth. Wanner and Goldluecke [34] used structure tensor and a convex optimization method to find the dominant slopes of the EPI and convert them to depth. Zhang et al. [35] used a spinning parallelogram operator to determine the disparity, which is given by the orientation of the parallelogram when the distance between the two regions enclosed in its two sides in an EPI is maximal.

More recently, Mishiba’s work [36] focused on devising a fast stereo matching-based depth-estimation algorithm for LF cameras. The main novelty lies in an offline cost volume interpolation, and in a weighted median filter which replaced the usual graph cut algorithm as global optimization solver, thus, increasing the speed of the overall algorithm.

### 2.2. Deep-Learning-Based Methods

An approach based on ML techniques addresses the depth-estimation problem from stereo pairs by employing learning-based methods for stereo matching. In [37], a supervised learning approach is proposed for predicting the correctness of stereo matches based on a random forest and a set of features about each pixel. In [38], the authors propose a framework which trains a model for matching cost computation in an unsupervised manner. In [39], the authors propose a deep-learning-based method that predicts the confidence level to improve the accuracy of an estimated depth map in stereo matching. This method was further improved in [40], where the confidence is estimated through a unified deep network, built based on the multi-scale patch processing approach that combines the confidence features extracted both from the matching probability volume and its corresponding  disparity.

Recently, several solutions based on ML techniques were developed to address the depth-estimation problem from 4D LF images by employing various DL-based methods. Feng et al. [22] proposed a two-stream Convolutional Neural Network (CNN) by training the network model using the pairs of block-size input patches extracted from the horizontal and vertical EPIs. Shin et al. [23] proposed a four-stream fully convolutional neural network (FCNN) where each stream was designed to process a block-size input patch extracted from a specific EPI (horizontal, vertical, main diagonal, or anti-diagonal). In our prior work [25], a neural network design is proposed to estimate each pixel depth by training network models using the input patches extracted from each of the following EPIs: horizontal, vertical, main diagonal, and anti-diagonal, and by further processing the four estimated depth maps to compute the final depth map. All these methods employ a pixel-wise strategy where the depth information of each pixel in the central view is estimated by inferring the patches from different EPIs containing the local neighborhood of the current pixel. Ma et al. [41] performed multi-scale convolutions to extract multi-scale features from the SAIs and obtain good estimations of the disparity in texture-less and reflective areas based on the estimations at object boundaries.

## 3. Proposed Method

**Figure 1:**
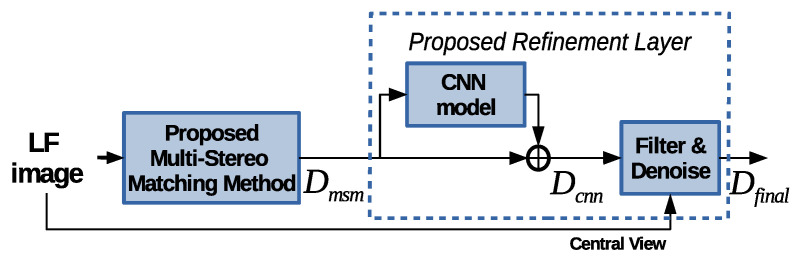
The scheme of the proposed depth-estimation method based on stereo matching and CNNs.

The baseline algorithm used for stereo matching was pioneered by Buades and Facciolo [30] and then reused by Navarro and Buades [31]. In our previous work [24], we have built on this algorithm by adapting it to operate on arrays of LF cameras. In this paper, we propose to first enhance our previous design in [24] to increase its robustness, and then employ a DL-based algorithm for residual error prediction to refine the final disparity estimation. Figure 1 depicts the scheme of the proposed method. The proposed multi-stereo matching (MSM) algorithm is first employed to compute the initial estimation, denoted by $D_{\mathrm{msm}}.$ The proposed CNN-based algorithm for residual error prediction is employed to compute additional details and to obtain the refined estimation, denoted by $D_{\mathrm{cnn}}.$ Finally, a post-processing algorithm is employed to compute the final estimation, denoted by $D_{\mathrm{final}}.$ In this paper, we focus on estimating the disparity information as it is well known that the depth information can be easily computed based on disparity, camera baseline, and focal length.

This section is organized as follows. Section 3.1 introduces the proposed MSM algorithm. Section 3.2 describes the proposed DL-based refinement algorithm.

### 3.1. Multi-Stereo Matching Method

The starting point of this work is represented by our algorithm from [24], where the proposed disparity estimation method based on a multi-scale multi-window approach was improved by employing the belief propagation [42] as the global energy minimization function. The method is capable of achieving sub-pixel accuracy, which is of critical importance when dealing with LF images that have very small disparity values. Furthermore, the method enforces the estimations to be reliable as unreliable pixels of each stereo pair are rejected, which leads to gaps that can be filled in based on estimations from other pairs. This approach is very well suited when dealing with LF images where multiple disparity estimations are to be fused together. Indeed, the accuracy of each disparity map is more important than its completeness as the missing pixels are likely to have an estimation in other stereo pairs.

In this paper, we propose to extend our previous work from [24] to improve the disparity estimation in flat and untextured areas. The following concepts are proposed: (i) remove the constraint that the two SAIs in the stereo pair must be aligned horizontally or vertically, and extend the set of stereo pairs to contain all available SAI pairs; and (ii) modify the disparity fusion algorithm to employ a weighted mean estimation based on the pixel confidence, instead of a median filter aggregation, and depending on the camera baseline.

#### 3.1.1. Neighborhood Window Selection

**Figure 2:**
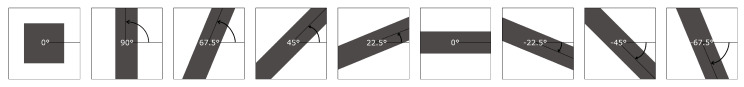
Windows with various orientations used in the stereo matching algorithm.

**Figure 3:**
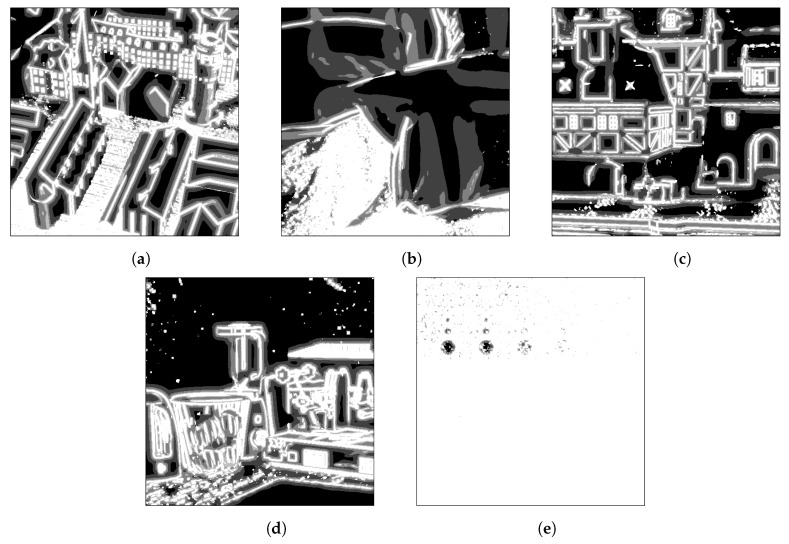
Illustration of the window size selection per pixel for five LF images from [43]: (**a**) *town*, (**b**) *pillows*, (**c**) *medieval2*, (**d**) *kitchen* and (**e**) *dots*. The smallest windows are marked in white and the largest ones in black.

To compute the disparity map corresponding to each stereo pair, eight different costs are computed for each pixel and disparity value using different neighborhood windows. These windows, which have different orientations, are depicted in Figure 2. By employing these windows instead of a regular squared window, the proposed multi-stereo method provides improved results in the areas close to objects boundaries and in the slanted regions where the selected window can align with the minimal disparity changes [31]. However, highly untextured areas remain difficult. To overcome this limitation, we introduce a threshold, denoted by τ, for the variance within the neighborhood inside the window. If the computed value is below τ, the size of the window is increased. This enforces the method to assure that enough information of the scene is available for finding overlapping patches in both stereo image, for each pixel *p* and for each of the eight windows. More exactly, four different window sizes are used in our implementation: 9×9, 17×17, 65×65, and 129×129. Figure 3 illustrates  the different windows selected for each pixel of the five LF images commonly used in the literature to compare the state-of-the-art methods for depth estimation, extracted from the dataset introduced in [43], where white marks the smallest window size and black marks the largest window size. As expected, uniformed areas such as the wall in the *kitchen* LF image use a big window size, while the very textured areas like the floor in the *town* LF image use a small window size. The same observation holds for the object boundaries, where very recognizable edges use the smallest windows.

The use of various window sizes improves the disparity estimation in large uniform areas. The threshold plays an important role: if it is too small, a 9×9 window will be assigned to all pixels which does not capture enough information about the scene in untextured areas; if it is too large, a 129×129 window will be used, which reduces the sharpness of object boundaries. Our threshold was empirically chosen and fixed for all images, to prove the robustness of the approach. Our experiments show that for the tested dataset, the PSNR increases with up to 0.35 dB and the number of reliable pixels increases with up to 3%.

In the case of stratified scenes such as *dots*, with a lot of noise, the variance is high everywhere in the image, thus limiting the impact of this threshold. Hence, here we propose to employ a DL-based algorithm to refine the initial estimation map by first detecting the cases where the proposed stereo matching method does not perform very well and then by computing the corresponding residual error to adjust the estimated disparity.

#### 3.1.2. Fusion Algorithm

LF cameras were invented as a different method to capture information of a given scene with the intention to improve its 3D reconstruction. Intuitively, it is clear that the more information of the incoming light should allow for better 3D reconstruction. In [31], only the horizontal and vertical SAIs, with respect to the SAI for which the disparity maps are computed for, are used. In contrast, the proposed method uses all available SAIs when necessary. However, to achieve such goal, we propose to extend our method to take into account the epipolar lines of each stereo pair, because the pairs that are not extracted from the same row or column are neither horizontally nor vertically registered. The epipolar lines are computed using the intrinsics and extrinsics matrices of each SAI. By adding the epipolar lines, the algorithm also gains the advantage of getting closer to reality, where two images captured by two different cameras are not likely to be registered.

The LF images in the 4D benchmark dataset from [43] consists of an array of 9×9 SAIs. Therefore, here we first apply the proposed stereo matching algorithm on all the available $9^{2}-1=80$ stereo pairs, where each pair contains the selected reference SAI (i.e., the corresponding view of the estimated disparity map) and one of the remaining SAIs, selected in turn. The algorithm computes 80 different disparity estimations for the reference SAI, which in our case is selected as the central view. The final disparity estimation is obtained by fusing the 80 disparity maps into one disparity map, $D_{msm}.$ In [24,31], the final disparity value for each pixel was computed as the median of the estimations. In this paper, we propose to carefully analyze the set of obtained estimations to find the best possible method to fuse them and compute the disparity maps.

**Figure 4:**
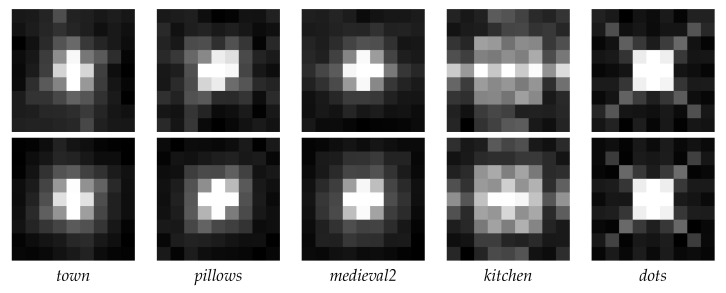
Visualization of the RMSE (1st row) and MAE (2nd row) of the disparity maps computed between the central SAI and each of the remaining 80 SAIs in the LF image, each pixel representing the error for one stereo pair.

**Figure 5:**
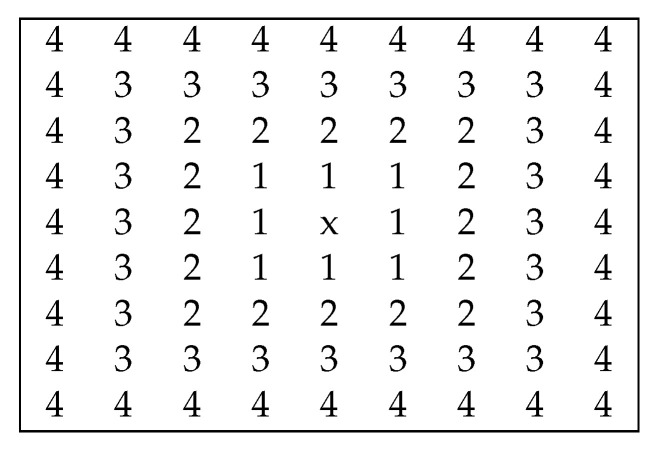
Representation of the baselines between SAIs relative to the central view (x), giving the order in which the final disparity map is to be filled in, starting from the largest ones.

Figure 4 depicts the Root Mean Square Error (RMSE) and the Mean Absolute Error (MAE) computed for each stereo pair between the estimated disparity map and the ground truth. Each image collects the RMSE (first row) and MAE (second row) on the positions of the stereo pair marked in Figure 5. One easily notes that the error decreases when the baseline between the stereo images increases. Moreover, the observations depicted in Figure 4 are consistent for all LF images in the dataset from [43], and not exclusively for this set of LF images. For the *kitchen* and *dots* LF images the visualizations look slightly different. Most probably, in the former, this is due to the reflective surfaces contained in the scene, and in the latter because of the high amount of noise. Regardless, the relationship between the baseline and estimation error remains the same. Therefore, we decided to give more importance to the disparity maps resulting from the SAIs with a bigger baseline. More exactly, we only use the disparities obtained from the inner SAIs if the estimations from the outer SAIs are not reliable and, thus, rejected, as explained in Section 3.1. These baselines are depicted in Figure 5. Please note that the figure does not express the actual distances between the SAIs, but merely provides a reference of the baseline distances as interpreted by the proposed algorithm.

In addition, the cost associated with each pixel, denoted by cost(i,j), was computed when estimating the disparity maps as the zero-mean sum of squared differences (ZSSD) between the patches in the two images, as expressed by Equation (2). Since this cost is inversely proportional to the similarity of the patches in the stereo pair, it is used here to compute the confidence level measurement of the found match, denoted by conf(i,j), and computed as follows:

(1) conf(i,j)=1−cost(i,j),

where cost(i,j) is the cost associated with pixel p=(i,j) int the first image for the chosen window *W* and disparity *d* leading the corresponding pixel *q* in the second image:

(2) $$C\left(p,q\right)=\frac{1}{\left|W\right|}\sum_{t\in W}\left|\left(u\left(p+t\right)-\bar{u_{W}\left(p\right)}\right)\right.{\left.-\left(v\left(q+t\right)-\bar{v_{W}\left(q\right)}\right)\right|}^{2}.$$

The output estimated disparity map of the proposed SM-based method, $D_{msm},$ is computed as the weighted mean of multiple estimated disparity maps *D* using the confidence metric as follows:

(3) $$D_{msm}\left(i,\;j\right)=\frac{1}{N}\sum_{k\in K}conf_{k}\left(i,\;j\right)D_{k}\left(i,\;j\right),$$

where

(4) $$N=\sum_{k\in K}conf_{k}\left(i,j\right),$$

and *K* is the set of estimated disparity maps to consider for a given pixel, based on their baselines and reliabilities.

The proposed fusion method, shown in Algorithm 1, combines *k* estimated disparity maps $D_{k}$ and their corresponding masks $m_{k}$ based on those two observations. For each pixel, the weighted mean of the reliable disparity maps is first computed using the largest available baseline, which has the value 4 in the case of an LF image represented as an array of 9×9 SAIs, for which the disparity map at the central location is computed. If not all pixels are filled in, due to the lack of a reliable estimation, the next available baseline, 3, is then used. This process is repeated until all pixels are filled in or no more disparity estimations are present. In general, due to the large amount of information available in an LF image, all the pixels are filled in. Otherwise, a simple inpainting technique is employed for the remaining pixels. Our tests show that the only remaining image holes are isolated pixel and not large patches and the values can easily be estimated from the surrounding pixels. Therefore, the remaining pixels are filled in based on their neighborhood’s disparity estimations, as the mean of the reliable values among its eight neighbors.
**Algorithm 1** Disparity maps fusion algorithm**Input:***k* disparity maps $D_{k}$, *k* reliability masks $m_{k}$, and *k* baselines $b_{k}$ **Output:** disparity map $D_{\mathrm{msm}}$
1:baseline = 42:**while** baseline > 0 **and** not all pixels filled in **do**3: **for all** pixels (i,j) in $D_{\mathrm{msm}}$ not yet filled in **do**4:  create empty vector *K* of estimations to consider5:  **for all** *k* **do**
6:   **if** $b_{k}$ == baseline **and** $m_{k}\left(i,j\right)==1$ **then**7:    add $D_{k}$ to vector *K*8:   **end if**
9:  **end for**
10:  $D_{\mathrm{msm}}\left(i,j\right)$ = weighted_mean(K,i,j)11: **end for**
12: baseline = baseline − 113:**end while**


### 3.2. Deep-Learning-Based Refinement

The estimated disparity map computed by the proposed MSM-based method is refined by employing a novel DL-based refinement method. The proposed pixel-wise DL-based algorithm is employed to process the local neighborhood information around the current pixel position and to estimate the corresponding residual error of the initial MSM-based estimation. Hence, the initial estimation is first adjusted with the CNN-based residual error prediction, and then further refined by employing a post-processing algorithm to compute the final estimation map.

The proposed training strategy is presented in Section 3.2.1, the proposed network architecture is described in Section 3.2.2, the loss function formulation is introduced in Section 3.2.3, while the final post-processing algorithm is outlined in Section 3.2.4.

#### 3.2.1. Training Strategy

The goal of the proposed DL-based algorithm is to process a small patch extracted from $D_{msm},$ and to evaluate the performance of the proposed MSM-based method by predicting its residual error. More exactly, the proposed DL-based algorithm uses the local context of the current pixel to detect the cases where the initially applied MSM-based method fails to provide a good disparity estimation, and then predicts the corresponding adjustment needed to correct the current estimation. The strategy was successfully applied in our previous work on lossless image compression [26,27]; however, in this paper, several design changes are required to refine the initial estimation. 

The input patch corresponding to the current pixel position, (i,j), is denoted by $X_{i,j}$ and contains the neighborhood of the current pixel, extracted from $D_{msm},$ by selecting all the rows and columns between the current position and a maximum distance of *b* pixels, as follows:

(5) $$X_{i,j}=\left[\begin{array}{ccccc} d_{msm}\left(i-b,\;j-b\right) & \ldots & d_{msm}\left(i-b,\;j\right) & \ldots & d_{msm}\left(i-b,\;j+b\right)\\ \vdots & \; & \vdots & \; & \vdots \\ d_{msm}\left(i,\;j-b\right) & \ldots & d_{msm}\left(i,\;j\right) & \ldots & d_{msm}\left(i,\;j+b\right)\\ \vdots & \; & \vdots & \; & \vdots \\ d_{msm}\left(i+b,\;j-b\right) & \ldots & d_{msm}\left(i+b,\;j\right) & \ldots & d_{msm}\left(i+b,\;j+b\right) \end{array}\right].$$

Here we set b=15 and generate input patches of size (2b+1)×(2b+1)=31×31, as our experiments show that this patch size offers a good performance vs. complexity trade-off.

Let us denote the ground truth map as $D_{gt}.$ For the current pixel position, the residual error of the proposed SM-based method, $\varepsilon_{i,j},$ is computed as follows:

(6) $$\varepsilon_{i,j}=d_{gt}\left(i,\;j\right)-d_{msm}\left(i,\;j\right).$$

A natural approach would require the target prediction of the proposed network, denoted by $y_{i,j},$ to be set as the residual error $\varepsilon_{i,j}.$ However, in the distribution of the residual error, one notes that for most of the samples, the target prediction is set with a value within a small range centered at 0. In such case, the neural network tends to ignore the large magnitude errors as not enough samples are available in the corresponding context so that the network can adjust its weights. Therefore, we propose to threshold the residual error using T=0.25 and set the target prediction as $y_{i,j}=-T$ for large negative errors and $y_{i,j}=T$ for large positive errors. More exactly, here, we choose to focus on predicting the large amount of small residual errors found in large image areas, since the high magnitude errors are sparse and their correction will have a small visual impact. Furthermore, we propose to quantize the residual error, $\varepsilon_{i,j},$ and set the target prediction, $y_{i,j},$ as follows:

(7) $$y_{i,j}=\left\{\begin{array}{cc} q\;\left\lceil \frac{\varepsilon_{i,j}}{q}\right\rceil , & \mathrm{if}\;\;\varepsilon_{i,j}<0,\\ 0, & \mathrm{if}\;\;\varepsilon_{i,j}=0,\\ q\;\left\lfloor \frac{\varepsilon_{i,j}}{q}\right\rfloor , & \mathrm{if}\;\;\varepsilon_{i,j}>0, \end{array}\right.$$

where $q=\frac{T}{N_{cls}}$ is the quantization step and $N_{cls}=100$ is the number of classes generated for the corresponding range. More exactly, we propose to assign each input patch to one of the $2N_{cls}+1=201$ available classes, where all the samples in a class have a single quantized residual error assigned. Based on this strategy, more samples are allocated to each class to create reliable contexts. One notes that the quantized residual error is rounded towards zero, so that the adjustment will introduce the smallest distortion.

In conclusion, the proposed neural network architecture (presented below) is trained based on the input patches $X_{i,j},$ extracted using Equation (5), and the target prediction $y_{i,j},$ set using Equation (7), and computes the residual error, denoted by $\tilde{\varepsilon }\left(i,j\right).$ Moreover, our tests have shown that a small improvement is obtained by quantizing $\tilde{\varepsilon }\left(i,j\right).$ Hence, $\tilde{\varepsilon }\left(i,j\right)$ is further processed using Equation (7) to obtain $\bar{\varepsilon }\left(i,j\right).$

Finally, the adjusted CNN map, $D_{cnn},$ is computed as follows:

(8) $$d_{cnn}\left(i,\;j\right)=d_{msm}\left(i,\;j\right)+\bar{\varepsilon }\left(i,\;j\right).$$

#### 3.2.2. Proposed Neural Network Design

**Figure 6:**
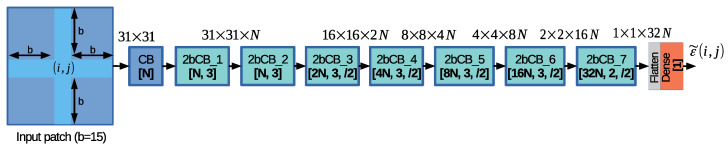
The proposed Depth Residual Error estimation Convolutional Neural Network (DRE-CNN) architecture design.

**Figure 7:**
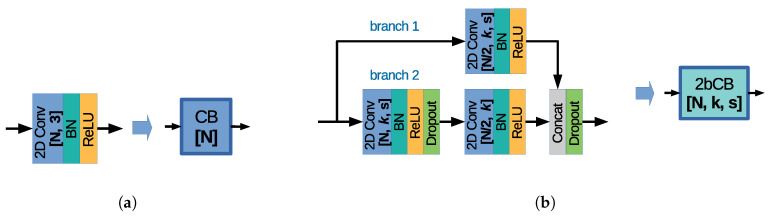
(**a**) Convolutional Block (CB) structure. (**b**) 2-branch Convolutional Block (2bCB) structure.

The proposed architecture is called Depth Residual Error estimation Convolutional Neural Network (DRE-CNN). Figure 6 depicts the DRE-CNN architecture design built based on two types of layer structure, the Convolution Block (CB) and the 2-branch Convolutional Block (2bCB), depicted in Figure 7a,b, respectively.

The CB block contains the following layer sequence: (i) a 2D convolution (2D Conv) layer with a 3×3 kernel; (ii) a batch normalization (BN) layer [44]; and (iii) a rectified linear unit activation function layer (ReLU). Please note that the parameters of the 2D Conv layers are set using the following notation “[N, k, s]”, where *N* denotes the number of channels, *k* denotes the k×k kernel size, and *s* denotes the stride. The default stride is s=(1,1) and it is omitted in the DRE-CNN design, while the stride s=(2,2) is denoted by s=/2.

The 2bCB block was inspired by the ResLB block proposed in our previous work [27]. 2bCB follows a two-branch strategy where “branch 1” is processing the input patch using one convolutional layer, while “branch 2” is processing the input patch using a sequence of two convolutional layers. Compared to the ResLB block [27], the 2bCB block proposes the following modifications: (1) introduces two Dropout layers [45] with a probability of 0.2 for setting the input samples to zero, one placed after the ReLU activation layer of the first 2D Conv layer in “branch 2”, and one placed at the end of 2bCB layer structure; and (2) replaces the addition layer with a concatenation (Concat) layer, resulting in halving the number of channels in 2D Conv layers before concatenation and reducing the network complexity.

The proposed DRE-CNN architecture contains one CB block, seven 2bCB blocks, and one dense layer, also known as fully connected layer. The CB block is equipped with *N* channels to process the input patch of size 31×31, and extracts the initial features. The following two 2bCB blocks are employed to further process the patches, while the remaining five 2bCB blocks are employed to reduce the patch resolution from 31×31 to 1×1 by employing a stride s=/2. Please note that the DRE-CNN design follows the general rule of doubling the number of channels whenever the path resolution is halved. The last 2bCB block contains 2D Conv layers with kernels of size 2×2. The last layer in the DRE-CNN architecture is a dense layer which contains one output as the network to estimate the final residual error, $\tilde{\varepsilon }\left(i,\;j\right).$ In this paper, we set N=16 and train models containing around 2.3 million (M) parameters. One notes that DRE-CNN takes advantage of both BN and Dropout concepts proposed in the literature to avoid overfitting.

#### 3.2.3. Loss Function Formulation

The loss function is computed based on the Mean Squared Error (MSE), and it employs $\ell_{2}$ regularization to avoid overfitting. Let us denote Θ as the set of all DRE-CNN model parameters, where $W_{i}\in \Theta$ are the trained weights; $X_{i}$ is the *i*th input patch of size 31×31; $y_{i}$ the corresponding target prediction set using Equation (7). Let F(·) be the function which processes $X_{i}$ using Θ to compute the predicted residual error as ${\tilde{\varepsilon }}_{i}=F\left(X_{i},\;\Theta \right).$ The loss function is formulated as follows:

(9) $$L\left(\Theta \right)=L_{MSE}+\lambda L_{L2},$$

where:

(a) $L_{MSE}$ is the loss term computed as the MSE between $y_{i}$ and ${\tilde{\varepsilon }}_{i}$ as follows:
  (10) $$L_{MSE}=\frac{1}{m}\sum_{i=1}^{m}{\left(y_{i}-{\tilde{\varepsilon }}_{i}\right)}^{2},$$
  where *m* is the number of samples in the batch.
(b) $L_{L2}$ is the $\ell_{2}$ regularization term computed as follows:
  (11) $$L_{L2}=\sum_{W_{i}\in \Theta }{∥W_{i}∥}^{2}.$$

In this paper, the DRE-CNN models are trained using $\lambda =10^{-2}.$

#### 3.2.4. Final Post-Processing

The output of the proposed DL-based algorithm, $D_{cnn},$ is then post-processed using the algorithm proposed in our previous work [25], where the estimated disparity is first filtered and then denoised based on a conventional algorithm. Hence, $D_{cnn}$ is first filtered twice using a mean filter with a 3×3 window, where the disparity values outside the $\left[\bar{d}-\tau ,\;\bar{d}+\tau \right]$ range are removed from the window, where τ=1 and $\bar{d}$ is the median value inside the window.

Finally, the disparity map is further processed to obtain the final disparity map, $D_{final},$ using the denoising algorithm proposed in [22] and available online. The algorithm uses a directional NonLocal Means (NLM) filter, where the neighborhood regularization term is defined based on the idea that the pixels with similar colors are encouraged to have similar depth values [46]. Therefore, the refinement of the disparity map is guided by the color information found in the corresponding central view (SAI) of the LF image. In the literature, various studies have proven that NLM can be efficiently used for image restoration and denoising [47], and depth map refinement [46,48].

## 4. Experimental Evaluation

### 4.1. Experimental Setup

The experimental and visual results are shown in comparison with the state-of-the-art depth-estimation methods proposed by: (a) Wang et al. [3]; (b) Williem et al. [32]; (c) Feng et al. [22]; and (d) Schiopu et al. [25]. The results for [3,32] are obtained by running the source codes, which were kindly provided by the authors of these methods. The numerical and visual results for [22] were extracted from the paper. The performance of these state-of-the-art methods is compared with the performance of the Proposed Multi-Stereo Matching method, denoted simply Proposed Stereo and presented in Section 3.1, and the proposed method depicted in Figure 1, denoted simply Proposed Method.

The experimental evaluation is performed on synthetic LF images [43]. The dataset contains 24 LF images, each represented as a grid of 9×9 SAIs, belonging to the sub-categories *additional*, *stratified,* and *training*, with available ground truth. Each SAI was captured using a resolution of 512×512.

The results of Proposed Method are obtained by first training a DRE-CNN model based on a set of LF images, called Training Set, and by inferring the model for the remaining LF images, called Test Set. In [22], the following training configuration is proposed: Training Set of 19 LF images; Test Set of following LF images: *town*, *pillows*, *medieval2*, *kitchen*, *dots*. Please note that from each LF image 512×512 = 262,444 samples are extracted, e.g., for the configuration of [22] a total number of 19 × 262,144 = 4,980,736 samples are extracted for training. In [25], other training configurations were proposed by randomly collecting a Training set of 20 LF images and a Test Set of the four remaining LF images, i.e., 5,242,880 samples are extracted for training. The Training Set is further divided into 15 LF images for model training and 4 LF images for model validation for the configuration from [22], and respectively 5 LF images in the configuration of [25]. Here, configurations C2 and C3 are selected to prove the robustness of Proposed Method. Therefore, three DRE-CNN models were trained, i.e., one for each training configuration. The Adam optimization algorithm [49] is applied because it is known as an improved procedure for adjusting the learning rate. Each model is trained during 40 epochs, using the learning rate of $2\times 10^{-4}$ and a batch size of 512 samples.

The performance is measured based on the MAE and RMSE metrics (where small values mark better results), and the structural similarity index measure (SSIM) [50] (where large values mark better results) computed between the ground truth and the estimated disparity map. Moreover, we introduce the Relative Gain (RG) (%) metric computed over the average results of a method *M* relative to Proposed Method as follows: $RG_{\mathrm{RMSE}}=\frac{RMSE_{M}}{RMSE_{\mathrm{Proposed}\;\mathrm{Method}}}-1,$ $RG_{\mathrm{MAE}}=\frac{MAE_{M}}{MAE_{\mathrm{Proposed}\;\mathrm{Method}}}-1,$ and $RG_{\mathrm{SSIM}}=1-\frac{SSIM_{M}}{SSIM_{\mathrm{Proposed}\;\mathrm{Method}}}.$

### 4.2. Experimental Results

Table 1 shows the depth-estimation results obtained for the training configuration of [22] for: the two traditional methods, [3,32]; the two DL-based methods, [22,25]; and the two proposed methods, Proposed Stereo and Proposed Method, where the bold values mark the best result. Please note that the conventional methods have different pre-processing and post-processing steps, including cost volume computation to produce the best possible results, therefore, only the final result is shown here for convenience. The average results show that the Proposed Method provides much better results compared to all other methods. One can note that based on the RMSE metric, the Proposed Method achieves an improved performance with 15.65% compare with [25] and 14.97% compared with Proposed Stereo. Based on the MAE metric, the Proposed Method achieves an improved performance with 43.62% compare with [25], and 22.69% compared with Proposed Stereo, which proves the efficiency of the proposed DL-based algorithm for residual error prediction. Moreover, based on the SSIM index, the Proposed Method achieves an improved performance with 12.12% compared with the state-of-the-art method based on conventional techniques [32], and an improved performance with 5.03% compared to the state-of-the-art method based on ML techniques [25].

**Figure 8:**
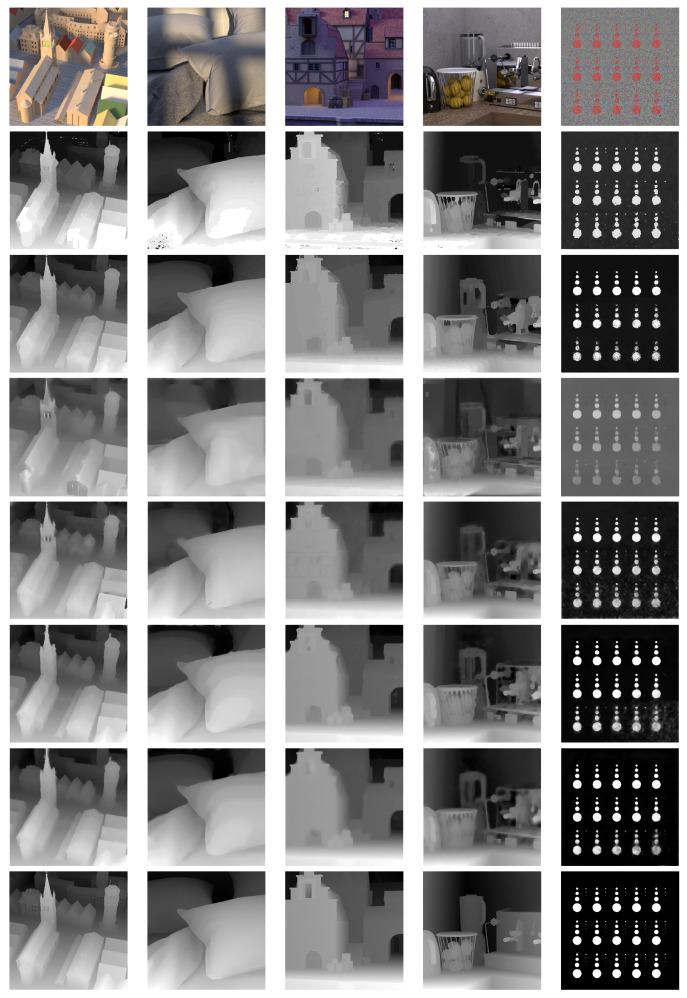
Visual results for the central view of 5 LF images in training configuration of [22], from left to right: *town*, *pillows*, *medieval2*, *kitchen*, and *dots*. (1st row) RGB images. (2nd row) Results for Wang et al. [3]. (3rd row) Results for Williem et al. [32]. (4th row) Results for Feng et al. [22]. (5th row) Results for Schiopu et al. [25]. (6th row) Results for our Stereo method. (7th row) Results for our Proposed method. (8th row) Ground truth disparity.

Figure 8 shows the visual results for the four state-of-the-art methods [3,22,25,32] and the two proposed methods.

One notes that the Proposed Method systematically improves the quality of the disparity map estimated by Proposed Stereo. E.g., in *town* (2nd column) the disparity around the church tower and the top of the tower is improved and the flat areas are smoothed, in *pillows* (3rd column) the pillow surface is smoothed, in *medieval2* (4th column) the entire disparity map is much sharper, and in *dots* (5th column) the background noise was removed completely. Moreover, one notes that the results of the Proposed Method look visually much better than the results of other methods. Figure 8 show the qualitative comparison of the five test LF images presented in Table 1. Due to the low resolution and continuous disparity maps in contrast to discrete disparity maps in conventional methods, the results of DL-based approaches look different than those of conventional methods, e.g., see the results of Schiopu et al. [25].

Table 2 and Table 3 shows the depth-estimation results for the training configuration C2 and C3, respectively, of [25], for the methods [3,25,32] and the two proposed methods, in MAE and SSIM. One notes that Proposed Method achieves the best overall results. Moreover, the DL-based refinement layer provides an improved performance with 9.77% in MAE and 6.60% in SSIM for configuration C2, and with 15.53% in MAE and 9.77% in SSIM for configuration C3, compared with Proposed Stereo.

**Figure 9:**
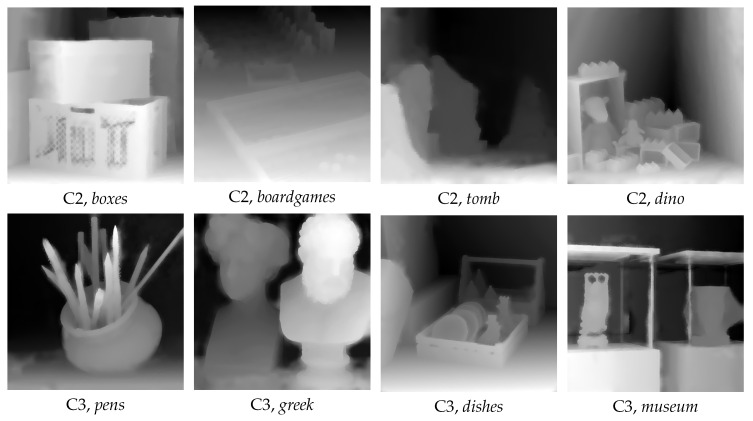
Disparity maps for the Proposed Method for configuration C2 and C3 of [25].

Figure 9 shows the visual results for the Proposed Method for the eight LF images of configurations C2 and C3. One notes that the Proposed Method provides: (i) sharp edges, e.g., see *pens, dino*; (ii) smooth areas, e.g., see *dishes*; and (iii) it is able to detect specific local features, e.g., see the pens in *pens*, and the toys in *dino*.

### 4.3. Ablation Study

Several design variations were tested in an ablation study focused on finding the best neural network design for residual error prediction. We analyze the impact of the following design decisions and concepts taken into account when building the proposed DRE-CNN architecture design: *(1)* employing the neural network as a classifier instead of a residual error predictor; *(2)* the efficiency of the 2bCB block compared with the ResLB block [27]; *(3)* the importance of the quantization step in the generation of the training data; *(4)* the influence of the input patch size on the method’s performance.

The first architecture variation studies the effect of employing the proposed network design as a classifier. More exactly, the DRE-CNN design was slightly modified by changing the number of output classes of the dense layer from 1 to $2N_{cls}+1=201$ and by adding a new SoftMax activation function as the last layer in the network, see Figure 6. This change requires that 4.45% more parameters (compared with DRE-CNN) are needed to train the weights of the extra 200 classes used in the last dense layer. In such case, the network will be employed to classify the input patch into a class, and the corresponding class index will select the quantized residual error computed by Equation (7). The obtained method is called *Classification design*.

The second architecture variation studies the efficiency of the 2bCB block compared with the ResLB block [27]. More exactly, the DRE-CNN design was modified by replacing all 2bCB blocks with corresponding ResLB blocks. Please note that in contrast to the concatenation layer in the 2bCB design, the addition layer in the ResLB design increases the number of parameters. This change introduced 173.69% more trainable parameters compared with the proposed DRE-CNN architecture. The obtained method is called *ResLB-based design*.

The third experiment studies the importance of pre-processing the training samples. More exactly, we propose to employ a smaller quantization step $q=\frac{T}{N_{cls}}$ in Equation (7), by using $N_{cls}=1000$ instead of $N_{cls}=100,$ see Section 3.2.1. Please note that in this case, only the training samples are modified, and no design change is applied to the proposed DRE-CNN architecture. The obtained method is called *Reduced quantization step*.

The last experiment studies how the input patch size influences the performance of the proposed method. Please note that in all other experiments, we set b=15 and generate input patches of size 31×31. In this experiment, we first propose to set b=11 and generate input patches of size 23×23. One notes that the network architecture remains the same, while the patches are processed at smaller resolutions. This reduces the runtime as the kernels are applied a lower number of times. The obtained method is called *Reduced patch size (b = 11)*. Secondly, we propose to further reduce the input patch size to less than a quarter by setting b=7 and generating input patches of size 15×15. In this case, the network architecture was slightly modified by removing the processing block *2bCB_7*, as shown in Figure 6, which further reduces the inference runtime. The obtained method is called *Quarter patch size (b = 7)*.

Table 4 shows the results for the two proposed methods and the experiments presented above. One notes that: *(i)* all DRE-CNN variations are still able to improve the performance of Proposed Stereo; *(ii)* DRE-CNN operates better as a predictor than as a classifier; *(iii)* the proposed 2bCB block structure provides important performance gain with a low complexity; *(iv)* training data pre-processing plays an important role in network training; *(v)* the inference runtime can be reduced by employing a smaller input batch; however, which will lead to a decreased performance.

### 4.4. Time Complexity

The proposed stereo estimation method requires around 9.45 min to process a stereo pair with the largest baseline; however, the runtime decreases further for more narrow baselines. Our experiments show that the proposed method can provide a good initial estimation with only 8 stereo pairs: 4 on the corners, 2 on the same row, and 2 on the same column. In this case, the performance of the proposed approach drops by only around 4%.

The proposed neural network is implemented in *Python* using the Keras open-source deep-learning library, and is running on machines equipped with *Titan Xp* Graphical Processing Units (GPUs). Table 4 shows the inference time for computing the refined estimation, $D_{cnn},$ for the different experiments proposed in the ablation study. The proposed DRE-CNN network requires around 23 hours to train one network model, and an average inference time of 47 ms for each batch of 512 input patches. Therefore, for each LF image, a total time of 512×0.047 s = 24.064 s is required to apply the CNN model. The experiments show that by halving the input patch resolution, the inference time can be reduced around 3.34 times, while the performance drops by around 3.18% in RMSE, 12.35% in MAE, and 0.22% in SSIM.

## 5. Conclusions

The paper proposed a novel depth-estimation method from LF images, which combines multi-stereo matching and ML techniques. A novel block-based stereo matching method is proposed to compute the initial disparity estimation by operating on any pair of two SAIs in the LF image. A novel DL-based method for residual error prediction is proposed to refine the initial estimation. A novel neural network architecture, DRE-CNN, is designed based on a more efficient layer structure, 2bCB. Experimental results on synthetic LF data demonstrate that the proposed framework outperforms quantitatively and qualitatively the existing state-of-the-art methods for depth estimation. 

## Figures and Tables

**Table 1:** Qualitative comparison in RMSE, MAE, and SSIM between computed and ground truth disparity maps for the training configuration of [22].

Method	Metric	LF Image					Average	RG
		*Town*	*Pillows*	*Medieval2*	*Kitchen*	*Dots*		
Wang et al. [3]	RMSE	0.3036	0.4239	0.3874	0.4518	0.2583	0.3650	215.19%
	MAE	0.2546	0.3737	0.3040	0.3432	0.2300	0.3011	500.54%
	SSIM	0.6897	0.7036	0.6448	0.5823	0.7307	0.6702	22.29%
Williem et al. [32]	RMSE	**0.0826**	0.0698	**0.0793**	0.2818	0.2110	0.1449	25.13%
	MAE	0.0410	0.0518	0.0341	0.2109	0.1170	0.0910	81.43%
	SSIM	0.8049	0.8327	0.8401	0.6044	0.7075	0.7552	12.12%
Feng et al. [22]	RMSE	0.1782	0.1403	0.1010	0.2673	0.2127	0.1799	55.34%
	MAE	0.1047	0.0971	0.0474	0.1605	0.1282	0.1076	114.60%
Schiopu et al. [25]	RMSE	0.1080	0.0717	0.0928	0.2593	0.1379	0.1339	15.65%
	MAE	0.0551	0.0433	0.0540	0.1456	0.0621	0.0720	43.62%
	SSIM	0.8175	0.9109	0.8649	0.7107	0.7913	0.8191	5.03%
Proposed Stereo	RMSE	0.1053	0.0756	0.0938	0.2418	0.1493	0.1332	14.97%
	MAE	0.0330	0.0309	0.0296	0.1238	0.0903	0.0615	22.69%
	SSIM	0.8273	0.9040	0.8850	0.6745	0.7962	0.8174	5.22%
Proposed Method	RMSE	0.0834	**0.0560**	0.0819	**0.2345**	**0.1232**	**0.1158**	anchor
	MAE	**0.0313**	**0.0302**	**0.0303**	**0.1199**	**0.0390**	**0.0501**	anchor
	SSIM	**0.8681**	**0.9232**	**0.9076**	**0.7263**	**0.8869**	**0.8624**	anchor

**Table 2:** Qualitative comparison in MAE and SSIM between computed and ground truth disparity maps for training configuration C2 of [25].

Method	Metric	LF Image				Average	RG
		*Boxes*	*Boardgames*	*Tomb*	*Dino*		
Wang et al. [3]	MAE	0.3643	0.3438	0.1942	0.3412	0.3109	363.29%
	SSIM	0.4748	0.6391	0.7212	0.5590	0.5985	26.70%
Williem et al. [32]	MAE	0.1488	0.0296	0.0403	0.0512	0.0675	0.54%
	SSIM	0.5995	0.8082	0.8086	0.7128	0.7323	10.32%
Schiopu et al. [25]	MAE	0.1599	0.0423	**0.0373**	0.0946	0.0836	24.52%
	SSIM	0.6245	0.8647	**0.8984**	0.7530	0.7852	4.00%
Proposed Stereo	MAE	0.1390	0.0225	0.0771	0.0560	0.0737	9.77%
	SSIM	0.5819	0.8614	0.8125	0.7950	0.7627	6.60%
Proposed Method	RMSE	**0.1346**	**0.0195**	0.0693	**0.0451**	**0.0671**	anchor
	SSIM	**0.6529**	**0.9305**	0.8455	**0.8374**	**0.8166**	anchor

**Table 3:** Qualitative comparison in MAE and SSIM between computed and ground truth disparity maps for training configuration C3 of [25].

Method	Metric	LF Image				Average	RG
		*Pens*	*Greek*	*Dishes*	*Museum*		
Wang et al. [3]	MAE	0.2428	1.2229	1.1973	0.1967	0.7149	548.54%
	SSIM	0.6107	0.4348	0.3544	0.7282	0.5320	32.46%
Williem et al. [32]	MAE	**0.0494**	0.3568	0.0721	**0.0552**	0.1334	21.02%
	SSIM	**0.7811**	0.5986	0.6345	**0.8215**	0.7089	10.00%
Schiopu et al. [25]	MAE	0.0807	0.4104	0.1290	0.1202	0.1851	67.89%
	SSIM	0.7715	0.6577	0.7727	0.7781	0.7450	5.42%
Proposed Stereo	MAE	0.1127	0.2516	0.0632	0.0819	0.1273	15.53%
	SSIM	0.6876	0.6438	0.7884	0.7230	0.7107	9.77%
Proposed Method	RMSE	0.0933	**0.2134**	**0.0571**	0.0771	**0.1102**	anchor
	SSIM	0.7769	**0.7358**	**0.8417**	0.7965	**0.7877**	anchor

**Table 4:** Study of the architecture and scheme variation of DRE-CNN.

Method	Nr. of Trainable Network Param.	Average			Average Relative Gain			Inference Time (s)
		RMSE	MAE	SSIM	${RG}_{\mathrm{RMSE}}$	${RG}_{\mathrm{MAE}}$	${RG}_{\mathrm{SSIM}}$	
Proposed Stereo	−	0.1332	0.0615	0.8174	14.97%	22.69%	5.22%	−
*Classification design*	2.4 M (+4.45%)	0.1182	0.0551	0.8528	2.11%	9.94%	1.11%	24.06
*ResLB-based design*	6.3 M (+173.69%)	0.1175	0.0558	0.8604	1.45%	11.36%	0.23%	25.09
*Reduced quantization step*	2.3 M	0.1200	0.0543	0.8587	3.63%	8.39%	0.43%	24.06
*Quarter patch size (b = 7)*	1.2 M	0.1195	0.0563	0.8605	3.18%	12.36%	0.22%	7.17
*Reduced patch size (b = 11)*	2.3 M	0.1173	0.0548	0.8609	1.31%	9.43%	0.17%	14.34
Proposed Method	2.3 M	**0.1158**	**0.0501**	**0.8624**	anchor	anchor	anchor	24.06
